# Stringent Abortion Laws and the Physician Struggle

**DOI:** 10.1097/og9.0000000000000056

**Published:** 2025-01-30

**Authors:** Chris Elizabeth Philip

**Affiliations:** Department of Obstetrics and Gynecology, Indiana University School of Medicine, Indianapolis, Indiana.

## Abstract

Restrictive abortion laws undermine evidence-based maternal care, forcing health care professionals to act against scientific standards and creating ethical dilemmas that compromise maternal health.

As a doctor working in obstetrics and gynecology, my journey has been shaped by a deep sense of empathy and a commitment to science, all while navigating the profound responsibility of advocating for women's health. In this narrative, I share my experiences across different countries, each with its own approach to abortion laws and their effects on women's access to care. I reflect on the rigid regulations that once constrained Ireland, the desperate pleas of Polish women making their way to the Czech Republic in search of assistance, and the reverberations of these challenges in the United States, particularly in the wake of the Supreme Court's decision to overturn *Roe v. Wade.*

My professional journey started in 2013, when I moved to Ireland to begin my residency training in obstetrics and gynecology. At the time, Ireland had some of the strictest abortion laws in Europe. Time and again, I stood by as women, desperately clinging to their pregnancies, were confronted with heartbreaking news. Fatal fetal anomalies, cancer during pregnancy, or life-threatening complications demanded interventions, yet legal constraints forced us to delay care. I watched women bleed and suffer, knowing that timely action could have saved them from pain and complications. The cruel reality of being unable to intervene as the flickering heartbeat of a nonviable fetus prolonged their agony left an indelible mark on me. It was a haunting reminder of how the assignment of personhood to a few cells can strip away humane care.

The devastation wasn't limited to these cases. Women who endured sexual assault came to us in fear, still reeling from trauma, only to discover they were pregnant. I delivered the news swiftly, leaving them with little space to process the horrors they had survived. I saw the fear, the hollow stares, and the weight of navigating these devastating circumstances while bound by laws that disregarded their suffering. It was a stark reminder that our medical system, tethered to legalities, often fails the very women it's meant to protect, forcing them into silence, secrecy, and exile for care they should have received at home.

The most heartbreaking cases were those of women for whom pregnancy posed an extraordinary risk to their survival. Women with chronic illnesses like cystic fibrosis or severe scoliosis, who had barely survived past pregnancies, returned with new pregnancies, often the result of contraceptive failure. They clutched their bellies in terror, fearing that, this time, their bodies might not make it. Yet, instead of offering them the care they needed, I had to tell them to travel abroad for termination, alone, financially strained, and burdened by a foreign health care system. On return, they often faced complications with no continuity of care, a glaring failure of a system that prioritizes restrictive laws over compassionate medical practice.

One poignant chapter in this narrative is the tragic case of Savita Halappanavar, a young dentist who lost her life in Ireland in 2012 due to complications from a septic miscarriage.^1^ Despite her repeated pleas for a termination, her doctors refused, citing Ireland's strict abortion laws. Savita's condition rapidly deteriorated, and she succumbed to septicemia, a preventable death that highlighted the cruel and inhumane consequences of inflexible legal constraints. Her case sparked a wave of public outrage and ignited intense debates about Ireland's abortion laws. In 2018, her story played a pivotal role in the successful campaign to repeal the Eighth Amendment, leading to the legalization of abortion in Ireland.^2^ Savita Halappanavar's legacy endures as a powerful testament to the need for laws that prioritize women's health and dignity.

Across the pond, after the U.S. Supreme Court's 2022 decision to overturn *Roe v. Wade*, a similar struggle for women's reproductive rights has emerged. The American Medical Association has strongly opposed abortion restrictions, calling them a violation of patient autonomy and a dangerous intrusion into the patient–physician relationship. They argue that physicians must make evidence-based decisions free from legal threats, because restrictive laws disproportionately harm patient safety, particularly in marginalized communities.^3^ However, without clear legal protection, doctors remain vulnerable to legal risks, creating an environment in which defensive medicine may overshadow optimal patient care.

At the heart of this dilemma are laws like Texas's S.B. 8, which bans abortion after about 6 weeks, deputizing private citizens to sue those involved in abortion care. This creates a culture of fear and incentivized “vigilantism,” setting a treacherous trap for health care professionals who must weigh their moral imperative to care for patients against the threat of legal peril. The devastating effects are clear: the year after the law took effect, Texas saw a 13% increase in infant deaths and a 23% rise in deaths from fetal conditions, compared with a 3.1% decrease nationwide.^4^ More alarmingly, maternal mortality in Texas rose by 56% from 2019 to 2022, disproportionately affecting Hispanic, Black, and White women, far surpassing the nationwide increase of 11%.^5^ These numbers mirror national trends, with studies showing that states with restrictive abortion laws face significantly higher maternal and infant mortality rates, with each added restriction increasing maternal deaths by 1.09 per 100,000 live births.^6^ These anti-choice laws are anything but pro-life. They trade evidence-based care for control, condemning women and infants to preventable deaths.

In the recent U.S. presidential debates, claims of women bleeding out in parking lots while waiting for abortion care were dismissed with disbelief. Yet, the reality is that miscarriages can be prolonged, and, without timely intervention, excessive bleeding can become life-threatening. Conservative management, which allows the body to expel tissue naturally, requires close monitoring because complications can escalate quickly. When the cervix is open and a fetal heartbeat persists, miscarriage is inevitable. However, laws that prioritize a fleeting electrical impulse over the health and future fertility of living women force dangerous delays, putting their well-being at unnecessary risk.

Much like Savita's case in Ireland, the United States has seen multiple tragic cases since the overturning of *Roe v. Wade*, where restrictive laws have led to fatal delays in miscarriage care. In Texas, Josseli Barnica and Nevaeh Crain both lost their lives after hospitals, fearing legal repercussions, withheld timely intervention for septic miscarriages.^7,8^ Similar delays claimed Amber Thurman's life in Georgia.^9^ These tragedies highlight the dangers of restrictive policies that lead health care professionals to practice defensive medicine, delaying urgent care when every second counts. Although it may seem logical to intervene only if the mother's life is in immediate danger, in obstetrics, situations can escalate in seconds. Hospitals in restrictive states often require health care professionals to seek legal or ethical clearance before intervening, slowing critical response times. Even when the path is clear, justifying urgency to legal authorities can be challenging. By the time permission is granted, vital moments have passed.

According to a sobering analysis by the United Nations, the dismantling of *Roe v. Wade* has undermined human rights in the United States.^10^ It highlights that denying essential health care, silencing health care professionals from discussing or referring to abortion care, and criminalizing pregnancy or health care specific to women constitute grave human rights violations. Furthermore, requiring young people to seek parental consent for health care and the disproportionate effect of abortion restrictions on people of color perpetuate inequality and violate human rights.^11^

During my medical studies in the Czech Republic, I witnessed the tragic reality of Polish women fleeing their country's draconian abortion laws, crossing borders for the care denied to them at home. In Poland, fear of legal backlash caused paralyzing delays where doctors could intervene only when a woman's life was in immediate danger. Meanwhile, Czech clinics treated these women with dignity and respect, giving them the agency Poland had stripped away. The contrast was stark: where one country silenced and endangered women, the other empowered them with the care they deserved.

The dilemmas faced by obstetricians and gynecologists are not simple; they exist on a spectrum of ethical complexities, often dictated by restrictive laws. These aren't just black or white; they are in fact shades of grey, and they extend far beyond, affecting every aspect of a woman's autonomy and health. Women's rights are human rights, and any system that infringes on these rights erodes the very foundation of ethical medical practice. We have a moral duty to stand firm, advocating for policies that ensure full reproductive care, including abortion. Protecting our patients requires that we demand not just empathy, but the legal autonomy to provide comprehensive care. Any attempt to intimidate or criminalize health care professionals for offering full, informed options, including abortion, is a direct assault on our ethical oath of nonmaleficence.

We cannot stand idle and wait for countless preventable tragedies like Savita Halappanavar's death. Her story, along with the others, should serve as a constant reminder of the deadly consequences of inaction. We must continually advocate for reproductive justice, drawing inspiration from how Savita’s legacy in Ireland has spurred significant change, and strive to achieve the same progress here. Our collective voice, amplified by evidence, compassion, and unyielding advocacy, can drive the change necessary to ensure that no woman is ever again forced into silence, exile, or danger in seeking the care she deserves. The future of reproductive rights hinges on our commitment to ensuring that health care remains a sanctuary, not a battlefield.
